# Plague Epizootic Dynamics in Chipmunk Fleas, Sierra Nevada Mountains, California, USA, 2013–2015

**DOI:** 10.3201/eid2604.190733

**Published:** 2020-04

**Authors:** Talisin T. Hammond, Kelly A. Liebman, Robert Payne, Helen K. Pigage, Kerry A. Padgett

**Affiliations:** University of California, Berkeley, California, USA (T.T. Hammond);; California Department of Public Health, Richmond, California, USA (K.A. Liebman, R. Payne, K.A. Padgett);; University of Colorado, Colorado Springs, Colorado, USA (H.K. Pigage)

**Keywords:** disease ecology, host–parasite, host–pathogen, Siphonaptera, *Tamias speciosus*, *Tamias alpinus*, *Yersinia pestis*, vectorborne disease, plague, chipmunk fleas, California, Yosemite National Park, Sierra Nevada, United States, bacteria, zoonoses, vector-borne infections, surveillance, fleas, rodents, infection prevalence

## Abstract

We describe *Yersinia pestis* minimum infection prevalence in fleas collected from *Tamias* spp. chipmunks in the Sierra Nevadas (California, USA) during 2013–2015. *Y. pestis*–positive fleas were detected only in 2015 (year of plague epizootic), mostly in *T. speciosus* chipmunks at high-elevation sites. Plague surveillance should include testing vectors for *Y. pestis*.

To better forecast vectorborne infection dynamics, characterizing disease cycles in both hosts and vectors is critical. The rate of infection of vector species can serve as a good indicator for risk during epizootic events, especially in areas with high human–wildlife overlap, but vectors are often poorly sampled. *Yersinia pestis*, the bacterium that causes plague, is carried by multiple flea species in western North America, where sciurids are often the primary reservoirs (*1*). Although human plague cases in this area are rare, in 2015, two cases were linked to exposures in Yosemite National Park, California, USA (*2*). In the investigation conducted to determine the source of these exposures, multiple *Y. pestis*–positive flea and rodent species were documented, and the lodgepole chipmunk (*Tamias speciosus*) was the host that was most frequently seropositive (*2*).

Plague surveillance in the western United States typically involves serologic testing of rodents and carnivores. Positive serologic results indicate prior plague activity. A *Y. pestis*–positive flea, however, indicates current plague transmission and is more likely to trigger control activities (*3*). Here, we sought to characterize *Y. pestis* infection in fleas of alpine (*T. alpinus*) and lodgepole (*T. speciosus*) chipmunks in Yosemite National Park and surrounding areas during 2013–2015. We focused on *T. speciosus* chipmunks because of their documented role in the 2015 epizootic (*2*) and on *T. alpinus* chipmunks because they co-occur with *T. speciosus* chipmunks (*4*) and little is known about their role in plague ecology. Our goals were to describe the proportion of *T. speciosus* and *T. alpinus* chipmunks harboring *Y. pestis*–positive fleas and the minimum infection prevalence of *Y. pestis* in fleas collected from these species across multiple sites and in years with and without known epizootic activity.

During June–October 2013–2015, we collected fleas from tagged chipmunks. Using a metal-pronged comb, we combed each animal 5 times down the dorsum, the tail, and each hind leg and placed collected fleas into vials containing 100% ethanol. These procedures were approved by the University of California, Berkeley, Animal Care and Use Committee (Berkeley, California, USA).

We identified key flea specimens (N = 122) (*5*–*9*) and then cleared, dehydrated, and mounted them on microscope slides (Denver Museum of Nature and Science accession nos. ZP.2000–176). For the remaining fleas, we microscopically observed and identified the species (*5*) using keys (*6*–*9*) and mounted some fleas as references. For each host, we pooled all conspecific fleas, which resulted in 162 pools (with 291 fleas total) from 121 *T. alpinus* chipmunks and 538 pools (with 1,096 fleas total) from 389 *T. speciosus* chipmunks (Appendix Table 1). We used molecular methods to detect *Y. pestis* DNA in flea pools (Appendix).

We found *Y. pestis*–positive fleas exclusively in 2015 at 5 of the 6 sites surveyed (Figure; Appendix Table 2). In 2015, 7.29% (14/192) of *T. speciosus* hosts carried >1 *Y. pestis*–positive flea. The minimum infection prevalence of *Y. pestis* in *T. speciosus* chipmunk–hosted fleas was 3.28% (assuming 1 positive flea per positive pool, 18 positive pools/548 total fleas in 280 pools tested). All 3 of the flea species (*Ceratophyllus ciliatus mononis*, *Eumolpianus eumolpi*, and *E. eutamiadis*) most commonly found on *T. speciosus* and *T. alpinus* chipmunks were found to be positive for *Y. pestis* (Appendix Table 1) (*10*). In 2015, a total of 5.13% (2/39) of *T. alpinus* hosts carried >1 *Y. pestis*–positive flea (Appendix Table 2). The infection prevalence (not minimum infection prevalence because each positive pool contained a single flea) of *Y. pestis* in *T. alpinus* chipmunk–hosted fleas was 2.47% (2 positive pools/81 total fleas in 50 pools tested). Unfortunately, these fleas were too damaged to identify morphologically, and molecular species identification was not possible.

**Figure:**
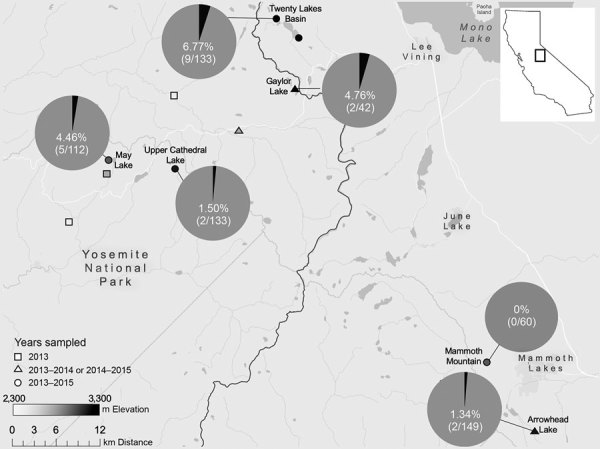
Minimum *Yersinia pestis* infection prevalence in fleas harbored by *Tamius speciosus* and *T. alpinus* chipmunks, by study site, Sierra Nevadas, California, 2013–2015. We visited sites for different numbers of years: 1 study year (2013 only), 2 study years, or all 3 study years. Plague prevalence was zero in 2013 and 2014, and map shows plague prevalence only in 2015. Pie charts show percentage of minimum infection prevalence (no. *Y. pestis* DNA–positive pools/no. fleas in pools tested). Sites without pie charts were either not visited in 2015 or had no flea pools collected there in 2015 because of low chipmunk prevalence. The irregular black line shows the eastern border of Yosemite National Park. Inset shows location of study sites in California. See Appendix Tables 1, 2 for more details on fleas tested.

*Y. pestis*–positive flea pools were detected at 5 of 6 high-elevation (2,650–3,200-m) study sites in 2015. Many of these sites are areas of high human activity, with popular hiking trails or established campgrounds. In 2015, plague risk assessments, including testing flea pools and rodent carcasses for *Y. pestis* DNA and rodent serology, also took place at lower elevation sites (1,778 ± 553 m) in and around the park; these surveys detected *Y. pestis* at 4 of 17 locations (*2*).

Altogether, our data indicate a dramatic shift in *Y. pestis* prevalence in fleas during a plague epizootic year in California. Our results support integrating flea testing, especially those at high-elevation sites, into regular surveillance.

Appendix More information about plague epizootic dynamics in chipmunk fleas, Sierra Nevada, California, USA, 2013–2015.
