# Supplementary material for: Plague Epizootic Dynamics in Chipmunk Fleas, Sierra Nevada Mountains, California, USA, 2013–2015
Source: Emerg Infect Dis. 2020 Apr;26(4):801–4. doi: 10.3201/eid2604.190733 (PMC7101107; doi:10.3201/eid2604.190733)
Supplement: Appendix — More information about plague epizootic dynamics in chipmunk fleas, Sierra Nevada, California, USA, 2013–2015. [file 19-0733-Techapp-s1.pdf]

Article DOI: https://doi.org/10.3201/eid2604.190733

Plague Epizootic Dynamics in Chipmunk Fleas, Sierra Nevada Mountains, California, USA, 2013–2015

Appendix

Molecular Methods

Fleas were suspended in 180 µL Buffer AL + 20 µL proteinase K (Qiagen, Hercules, CA) in cryovials containing three 2.8 mm ceramic beads. Pools were then homogenized using a Bead Ruptor (Omni Inc, Kennesaw, GA). DNA was extracted using Qiagen DNeasy Blood and Tissue Kit per the manufacturer's protocol (Qiagen, Hercules, CA). DNA samples were eluted to a final volume of 200 µL. A real-time PCR protocol using TaqMan primers and probe targeting the *caf1* gene was used to detect *Y. pestis* DNA (*1*). The volume of nucleic acid for each PCR reaction was 1 µL. Each 20 µL reaction included the sample and the master mix, which was a combination of 10 µL Bio-rad SsoAdvanced Universal Probes Supermix, 8.4 µL Sterile Water, 0.2 µL primer F, 0.2 µL Primer R, and 0.2 µL probe. Cycling conditions were as described in (*1*). Samples were run in singlicate. Any samples that were positive for CAF1 in initial testing were included in a second confirmatory PCR with CAF-1, YPO and LCRV primers/probes. Samples were considered positive at CT values of <38.

Appendix Reference

1. Liu J, Ochieng C, Wiersma S, Ströher U, Towner JS, Whitmer S, et al. Development of a TaqMan array card for acute febrile illness outbreak investigation and surveillance of emerging pathogens including Ebola virus. J Clin Microbiol. 2016;54:49–58. PubMed https://doi.org/10.1128/JCM.02257-15

Appendix Table 1. Number of flea pools tested in each year, arranged by host and flea species*

Year	Host species (no.)	*C. ciliates mononis*	*E. eumolpi*	*E. eutamiadis*	*Oropsylla montana*	*Aetheca wagneri*	Species unknown
2013	*T. alpinus* (34)	9 (0)	39 (0)	1 (0)	-	-	1 (0)
	T. speciosus (76)	58 (0)	26 (0)	21 (0)	1(0)	-	5 (0)
2014	*T. alpinus* (48)	22 (0)	36 (0)	3 (0)	-	-	1 (0)
	T. speciosus (121)	104 (0)	30 (0)	12 (0)	-	-	1 (0)
2015	*T. alpinus* (39)	9 (0)	34 (0)	1 (0)	-	-	6 (2)
	T. speciosus (192)	156 (7)	74 (7)	36 (3)	-	1 (0)	13 (1)
Totals		358 (7)	239 (7)	74 (3)	1 (0)	1 (0)	27 (3)

*For each flea species, the number of *Y. pestis*-positive pools is shown in parenthesis. Mean (±SE) pool size was 1.80 ± 0.11 fleas for *T. alpinus* and 2.04 ± 0.11 for *T. speciosus.*

Appendix Table 2. 2015 Study site locations and numbers of fleas, flea pools, hosts, and information regarding *Y. pestis* status*

2015 Sites	Latitude	Longitude	Elevation, m	Host species	No. fleas (no. pools, no. hosts)	No. pools *Y. pestis*-positive (no. hosts)	Minimum *Y. pestis* infection prevalence in fleas	% Hosts with at least 1 *Y. pestis*-positive flea
AL	37.584044	–118.97638	2970	*T. speciosus*	149 (85, 58)	2 (2)	1.34%	3.45%
CL	37.838859	–119.41795	2960	*T. alpinus*	47 (27, 21)	0 (0)	0%	0%
				T. speciosus	86 (41, 29)	2 (2)	2.33%	6.90%
GL	37.91332	–119.26717	3190	*T. alpinus*	11 (9, 6)	2 (2)	18.18%	33.33%
				T. speciosus	31 (14, 9)	0 (0)	0%	0%
MA	37.651181	–119.03418	2650	*T. speciosus*	60 (41, 33)	0 (0)	0%	0%
ML	37.845177	–119.49606	2870	*T. alpinus*	19 (10, 8)	0 (0)	0%	0%
				T. speciosus	93 (44, 27)	5 (3)	5.38%	11.11%
TL	37.981377	–119.2918	3110	*T. alpinus*	4 (4, 4)	0 (0)	0%	0%
				T. speciosus	129 (55, 36)	9 (7)	6.98%	19.44%

*AL, Arrowhead Lake; CL, Cathedral Lake (upper); GL, Gaylor Lakes; MA, Mammoth; ML, May Lake; TL, Twenty Lakes Basin.
